# Prevalence and Factors Associated with Neonatal Hypothermia in Sub-Saharan Africa: Systematic Review and Meta-Analysis

**DOI:** 10.3390/jcm15051818

**Published:** 2026-02-27

**Authors:** Hundessa Daba Nemomssa, Frederick Bossuyt, Bjorn Vandecasteele, Herbert De Pauw, Netsanet Workneh Gidi, Pieter Bauwens

**Affiliations:** 1 Center for Microsystems Technology (CMST), IMEC, Ghent University, 9000 Gent, Belgium; 2 School of Biomedical Engineering, Jimma Institute of Technology, Jimma University, Jimma 378, Ethiopia; 3 Department of Health Behavior and Society, Jimma Institute of Health, Jimma University, Jimma 378, Ethiopia; netsanet.workneh@ju.edu.et

**Keywords:** neonates, hypothermia, prevalence, risk factors, sub-Saharan Africa

## Abstract

**Background/Objectives**: Neonatal hypothermia remains a significant contributor to neonatal mortality and morbidity mainly in low and middle-income countries, such as those in sub-Saharan Africa. The objective of this systematic review and meta-analysis is to assess the prevalence of neonatal hypothermia and its risk factors in sub-Saharan Africa. **Methods**: The Preferred Reporting Items for Systematic Review and Meta-Analysis statement (PRISMA) guideline was used to search databases (PubMed, Scopus, Cocrane library and Google Scholar) for studies reporting both the prevalence and factors associated with neonatal hypothermia in sub-Saharan Africa. We have included cross-sectional, cohort and descriptive studies published between 1 June 2014 and 31 May 2024. The Joanna Briggs Institute (JBI) quality appraisal checklist was used for the appraisal of studies. Subgroup analysis was conducted by country, study design and population. A total of 21 articles with 12,803 participants from 9 countries were included in the analysis. **Results**: The pooled prevalence of neonatal hypothermia was 55.39% (95% CI: 48.52, 62.25). Preterm birth (odds ratio (OR): 3.49; 95% CI: 1.98–6.16), low birth weight (OR: 3.56; 95% CI: 2.36–5.39), no skin-to-skin contact (OR: 1.31; 95% CI: 0.55–3.13), lack of resuscitation (OR: 2.56; 95% CI: 1.75–3.76), delayed initiation of breast feeding (OR: 2.38; 95% CI: 1.57–3.61), admission during cold season (OR: 1.80; 95% CI: 1.33–2.44), home delivery (OR: 1.94; 95% CI: 1.51–2.50) and early bathing (OR: 3.03; 95% CI: 0.98–9.38) were the factors significantly associated with neonatal hypothermia. **Conclusions**: The observed high prevalence of hypothermia was associated with physiological, behavioral and environmental factors.

## 1. Introduction

The neonatal period, encompassing the first 28 days after birth, is the most critical time for newborn survival, with a significant portion of child mortality occurring during this stage [1,2,3]. Despite the global decline of neonatal mortality over the decades, millions of newborns continue to die annually due to various health complications [4,5,6,7]. In 2022 alone, the World Health Organization (WHO) reported 2.3 million neonatal deaths [3]. Most of these fatalities occur in low- and middle-income countries, particularly in Sub-Saharan Africa, which accounts for 38% of the global newborn mortality [3,4,6,7]. Many of these deaths occur in the first week, with nearly one million deaths occurring on the first day [8]. The problem persists as a result of inadequate newborn care, risk identification, and risk management [5,9].

Neonatal hypothermia, defined as a drop in core body temperature below 36.5 °C [10], contributes significantly to neonatal mortality and morbidity, especially in resource-limited settings such as sub-Saharan Africa [11,12,13,14,15,16]. Unlike adults, neonates lack effective shivering mechanisms and rely mostly on non-shivering thermogenesis via brown adipose tissue. Their high surface-area-to-body-mass ratio, thin skin, little subcutaneous fat, and immature vasomotor control make them susceptible to rapid heat loss through evaporation, radiation, convection, and conduction [13]. Adults, on the other hand, regulate their body temperature through shivering, behavioral responses, and improved insulation [14]. Clinically, neonatal hypothermia may manifest as cool extremities, lethargy, poor feeding, hypotonia, respiratory instability, hypoglycemia, and metabolic acidosis, and severe cases can progress to cardiorespiratory failure, sepsis, and death [10,15]. The World Health Organization classifies neonatal hypothermia into mild (36.0–36.4 °C), moderate (32.0–35.9 °C), and severe (<32.0 °C) categories, with increasing risk of adverse outcomes across severity levels [16].

Multiple biological, environmental, and healthcare-related factors increase the risk of neonatal hypothermia, including prematurity, lack of skin-to-skin contact after delivery, low birth weight, delayed initiation of breastfeeding, resuscitation at birth, low environmental temperatures, nighttime deliveries, low socio-economic status of families [17], low APGAR scores, and inadequate knowledge of thermal care among healthcare workers [18,19].

The global prevalence of neonatal hypothermia at the hospital level was reported to vary between 32% and 85%, and the peak prevalence of 92% was reported for home deliveries [20,21]. The prevalence of hypothermia in sub-Saharan Africa varies by country and region. To improve the survival of newborns in sub-Saharan Africa, it is important to know how common neonatal hypothermia is and what factors put babies at risk for it.

Despite several studies reporting on the prevalence and risk factors of neonatal hypothermia across various sub-Saharan African countries, a comprehensive synthesis of the available evidence is lacking. Therefore, the objective of this systematic review and meta-analysis is to determine the overall prevalence of neonatal hypothermia and to identify the key factors associated with it in sub-Saharan Africa.

## 2. Materials and Methods

### 2.1. Reporting

The protocol for this systematic review and meta-analysis was registered with PROSPERO under the registration number CRD42024593317, which can be accessed at: https://www.crd.york.ac.uk/prospero/display_record.php?ID=CRD42024593317, accessed on 11 October 2024. The results of this review were reported based on the Preferred Reporting Items for Systematic Review and Meta-Analysis statement (PRISMA) guideline [22]. The completed PRISMA 2020 checklist is provided in the Supplementary Materials (‘Table S1: PRISMA checklist’).

### 2.2. Search Strategies

This review considered research articles that provide data on the prevalence and factors associated with neonatal hypothermia in sub-Saharan Africa. A comprehensive search was conducted across several databases, including Scopus, PubMed, the Cochrane Library, and Google Scholar. The main search terms and phrases included “neonatal”, “infant”, “newborn”, “hypothermia” and “sub-Saharan Africa”. Various Boolean operations were employed to refine the search results. For the advanced PubMed search, the following strategy was used: [(Prevalence OR magnitude) AND (causes OR determinants OR associated factors OR predictors) AND (neonatal [MeSH Terms] OR infant OR newborn) AND (hypothermia [MeSH Terms] OR cold stress OR low body temperature) AND (Sub-Saharan Africa) OR developing countries].

### 2.3. Study Selection and Screening

Studies were imported into Rayyan AI to remove duplicates. Two investigators independently screened the titles and abstracts before accessing the full-text articles, using pre-defined criteria for further evaluation. Any disagreements were settled according to the established guidelines.

### 2.4. Inclusion and Exclusion Criteria

We included observational studies (cross-sectional, cohort, and descriptive studies) that reported the prevalence of neonatal hypothermia and associated risk factors. These studies had to be published in English between 1 June 2014, and 31 May 2024, within sub-Saharan Africa. We excluded anonymized reports, editorials, qualitative research, and citations lacking an abstract or full text in the evaluation.

### 2.5. Quality Assessment

The Joanna Briggs Institute (JBI) quality appraisal checklist was used by two independent authors to assess the quality of the studies. Studies that received a score of 4 or higher were considered low risk or good quality. Studies scoring 3 or lower were seen as high risk or of poor quality.

### 2.6. Data Extraction

The two reviewers extracted relevant data from the included studies to an Excel spreadsheet. This included the following characteristics: author, year, country, study design, sample size, definition of hypothermia, type of study population, prevalence of hypothermia, and associated factors. Any discrepancies in data extraction were resolved through discussion.

### 2.7. Statistical Analysis

The prevalence of neonatal hypothermia was pooled using a random-effects meta-analysis model. This method accounts for both within-study and between-study variability, giving a clearer estimate of the overall prevalence. Heterogeneity, which refers to differences in prevalence estimates across studies, was assessed using the I2 statistic. Conventionally, I2 values of 25%, 50%, and 75% are seen as low, moderate, and high heterogeneity, respectively. To explore potential sources of heterogeneity, subgroup analyses were conducted based on the country, study design, and population of included studies. Sensitivity analysis determined the impact of a single study on the total estimate. For the analysis of risk factors linked to neonatal hypothermia, the researchers used a random-effects meta-analysis model to pool the reported odds ratios (ORs). All analyses were performed using R software version 4.5.1.

## 3. Results

### 3.1. Literature Screening Results

**Figure 1:**
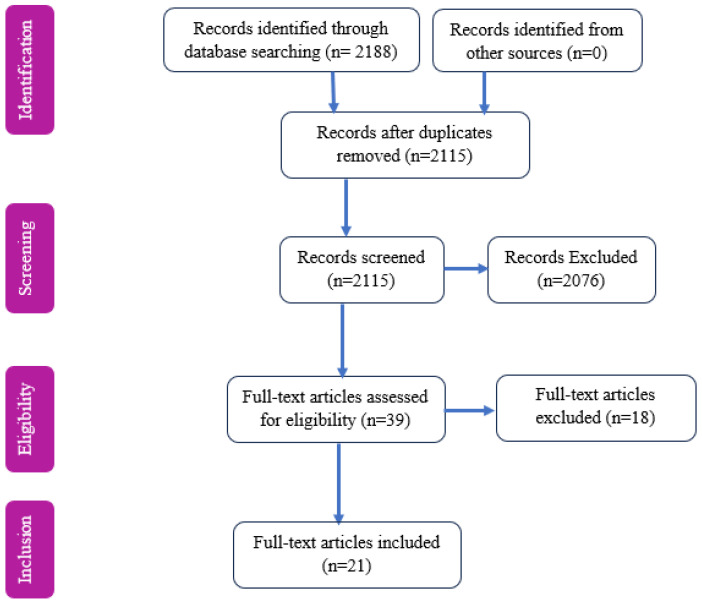
PRISMA flow diagram showing the article selection process.

A total of 2188 articles were initially retrieved, comprising 1874 from PubMed, 56 from Scopus, 18 from the Cochrane Library and 240 from Google Scholar. Following the application of rigorous inclusion and exclusion criteria encompassing study design, participant demographics, and relevant outcomes, 2115 articles were screened based on their titles and abstracts, followed by a full-text review of 39 studies. Ultimately, 21 articles with 12,803 participants met the inclusion criteria and were selected for this review (Figure 1).

### 3.2. Characteristics of Included Studies

The characteristics of the 21 studies included in this review are summarized in Table 1. Of the selected studies, eight are from Ethiopia, four from South Africa, two each from Nigeria and Ghana, and one each from Tanzania, Rwanda, Malawi, Kenya, and Uganda. Seventeen studies are cross-sectional, two are cohort studies, and two are descriptive studies. Additionally, the populations in these studies include fourteen conducted on all neonates, three on preterm neonates, two on low-birth-weight neonates, one on preterm low-birth-weight neonates, and one on neonates born through the caesarean section. All studies were published between 2018 and 2024, and all defined hypothermia as an axillary temperature below 36.5 °C.

### 3.3. Meta-Analysis

#### 3.3.1. Prevalence of Neonatal Hypothermia

**Figure 2:**
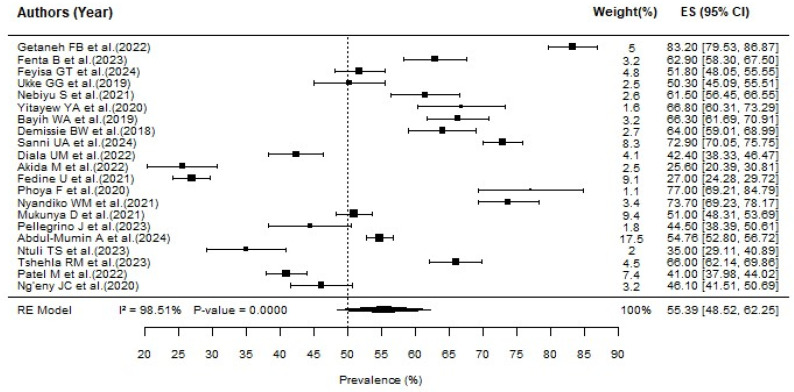
Pooled prevalence of neonatal hypothermia in Sub-Saharan Africa [10,17,23,24,25,26,27,28,29,30,31,32,33,34,35,36,37,38,39,40,41].

The prevalence of neonatal hypothermia in the included studies ranges from 25.6% [32] to 83.2% [23]. The pooled prevalence of neonatal hypothermia in Sub-Saharan Africa is 55.39% (95% CI; 48.52–62.25; I^2^ = 98.51%; *p* < 0.001) (Figure 2). This high level of heterogeneity indicates significant variability among the studies, suggesting that factors such as geographic location, study design, and population characteristics may influence prevalence rates.

#### 3.3.2. Subgroup Analysis

Subgroup analysis was conducted based on country, study design, and population. Accordingly, the prevalence of neonatal hypothermia was found to be 63.38% in Ethiopia, 50.05% in Ghana, 73.70% in Kenya, 77.00% in Malawi, 57.69% in Nigeria, 27% in Rwanda, 47.10% in South Africa, 25.6% in Tanzania, and 51% in Uganda (Table 2). Based on study design, the pooled prevalence of neonatal hypothermia was 73.13% in cohort studies, 55.3% in cross-sectional studies and 38.55% in descriptive studies. Based on the study population, the prevalence of neonatal hypothermia was 73.07% in preterm neonates, 55.48% in neonates, 45.52% in low-birth-weight neonates, 41.0% in neonates born through caesarean section, and 35.0% in preterm low-birth-weight neonates.

#### 3.3.3. Publication Bias

**Figure 3:**
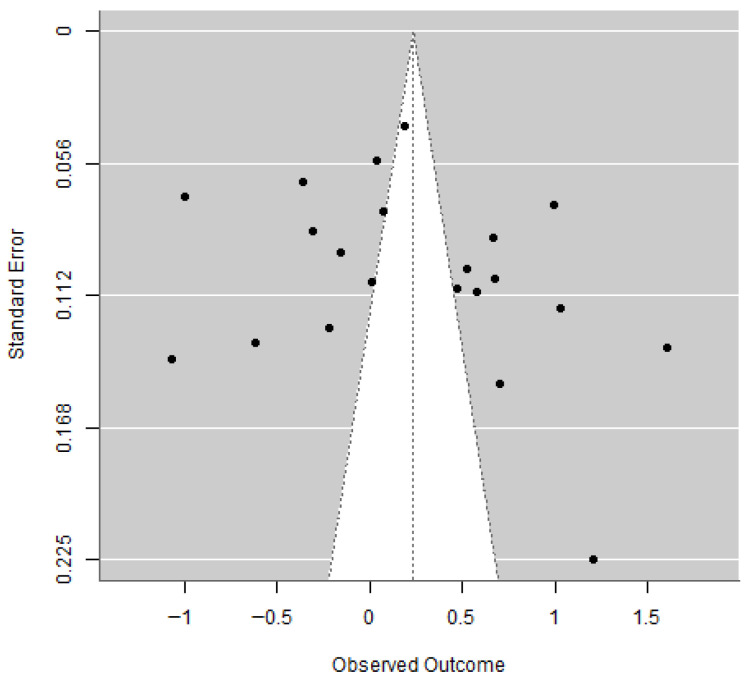
Funnel plot of publication bias.

The funnel plot (Figure 3) shows an approximately symmetrical distribution of studies. The Egger’s regression test value was 0.9123, and the *p*-value was 0.3731, which is greater than 0.05, indicating no statistically significant evidence of publication bias. However, given the substantial between-study heterogeneity, these findings should be interpreted with caution, as heterogeneity may reduce the reliability of funnel plot–based methods.

To further assess potential small-study effects, a trim-and-fill analysis was performed. The procedure did not impute any missing studies, and the trim-and-fill–adjusted pooled prevalence remained at 55.39%, identical to the original estimate. This suggests that publication bias is unlikely to have materially influenced the pooled prevalence.

A leave-one-out sensitivity analysis showed that the pooled prevalence of neonatal hypothermia remained stable following sequential exclusion of individual studies, with recalculated pooled estimates ranging from 53.01% to 57.11%. Excluding the study reporting the highest prevalence (83.2%) resulted in only a minimal change in the pooled estimate, indicating that no single study exerted a disproportionate influence on the overall prevalence. These findings support the robustness and reliability of the pooled prevalence estimate despite substantial heterogeneity.

#### 3.3.4. Factors Associated with Neonatal Hypothermia

Meta-analysis was conducted for factors reported by more than two articles using a random-effects model. From this analysis, eight factors (preterm birth, low birth weight, no skin-to-skin contact, lack of resuscitation, delayed initiation of breast feeding, admission during the cold season, home delivery and early bathing) were significantly associated with an increased incidence of neonatal hypothermia. The pooled OR of these factors together with the heterogeneity test results (I^2^ and *p*-value) are summarized in Table 3. In addition, forest plots for pooled risk factors are provided in Supplementary Figures S1–S8.

## 4. Discussion

Neonatal hypothermia is one of the leading causes of death among newborns in sub-Saharan Africa countries [42]. This systematic review and meta-analysis gives a thorough evaluation of the prevalence and determinants of neonatal hypothermia in the region. Similarly, this review presents updated pooled estimates of neonatal hypothermia in sub-Saharan Africa by providing useful information for health planners, policymakers, and the community.

This systematic review and meta-analysis showed that the pooled prevalence of neonatal hypothermia in sub-Saharan Africa was 55.39% (95% CI; 48.52–62.25), which indicates a serious public health problem that needs urgent attention. The overall prevalence of neonatal hypothermia in the current review was higher compared to the global prevalence of 52.5% reported by Ruan J. et al. [43] and the prevalence rate of 26.8% in Canada [44]. However, the magnitude was slightly lower than those found in studies conducted in East Africa and Ethiopia, where the reported prevalence was 57.2% and 60.96%, respectively [20,45]. Compared with Beletew et al. [20], exclusively focusing on East Africa regions, our current review extends geographic representation to nine countries in sub-Saharan Africa, with additional recent studies included. Unlike Ferede et al. [45], whose work focused only on Ethiopia, the generalization of our work extends far beyond that single region. Discrepancies in pooled prevalence data exist due to differences in the targets of the studies, as well as the inclusion of preterm-specific datasets in previous systematic reviews. Our study will provide updated aggregated data and broadened analysis of risk factors at the regional scale.

Limited research has indicated the prevalence of neonatal hypothermia at health facilities in Asia, with Nepal showing 64% and Sri Lanka showing 63%, both of which are higher than the pooled prevalence rate identified in the current study [46]. This is due to variations in the settings, sample size, and duration of the studies. Additionally, the majority of studies featured in this review took place in neonatal intensive care units, where admitted infants constitute a higher-risk patient group. As a result, the combined prevalence probably inflates hypothermia rates in the overall neonatal population, encompassing healthy newborns born at home or discharged early. This bias in selection restricts the generalizability at the population level.

Analysis of the sub-groups by country revealed that the highest occurrence of neonatal hypothermia (77%) was in Malawi [34]. Nevertheless, it is essential to recognize that the data from Malawi might not be entirely representative, as it originates from a single study. Significantly, according to subgroup analysis based on the study population, preterm neonates had the highest prevalence of neonatal hypothermia, at 73.07%. This is due to a large surface area-to-body mass ratio, less subcutaneous fat, less brown fat, and underdeveloped organs, which hinder effective body temperature regulation [10,28,47].

Our pooled risk-factor analysis identified several significant determinants spanning physiological, behavioral, environmental, and delivery-related domains. In the random-effects model pooled estimate, preterm birth (OR 3.49) and low birth weight (OR 3.56) were significantly associated with neonatal hypothermia. Neonates with delayed initiation of breastfeeding (OR 2.38), no resuscitation at birth (OR 2.56), home delivery (OR 1.94), and admission during the cold season (OR 1.80) were also at a higher risk. These findings highlight the relationship between clinical vulnerability, care giving practices, environmental exposure, and health system capacity. These findings align with previous studies, reinforcing the multifaceted nature of neonatal hypothermia [20,43,48].

From the random-effects model estimate, the odds of neonatal hypothermia were higher among preterm neonates when compared with neonates delivered after full term. A possible reason might be the vulnerability of preterm neonates to high evaporative heat loss due to their large surface area-to-mass ratio, lack of insulating subcutaneous fat, and immature skin deficient in keratin [49]. Preterm infants also have very limited heat generation ability due to underdeveloped organs [50]. Additionally, limited evidence from low- and middle-income countries indicates that many preterm infants are not kept warm at birth due to insufficient resources and expertise [26,30]. Similarly, the odds of neonatal hypothermia were higher among neonates with low birth weight compared to neonates born with normal weight. The odds of hypothermia were also high among the neonates with delayed initiation of breastfeeding. This could be due to the high calories that newborns get from breast milk, which helps shield them from hypothermia. Moreover, breastfed neonates are more likely to have had skin-to-skin contact with their mothers, which helps safeguard them from hypothermia [51].

Behavioral factors, such as the absence of skin-to-skin contact and immediate bathing, revealed no notable or borderline aggregated effect, with broad confidence intervals signifying imprecision and uncertainty. While these activities are biologically plausible contributors to hypothermia, the existing pooled evidence is unclear, and more high-quality research is required to determine their separate impacts. The odds of neonatal hypothermia were higher among neonates who didn’t have skin-to-skin contact with the mother compared to neonates who had skin-to-skin contact after delivery. A possible reason might be the warm chain principle that there is no heat transfer from the mother to neonates [29]. The pooled effects of individual risk factors are presented in Supplementary Figures S1–S8 and a summary table (Table 4), which illustrate the magnitude, direction, and consistency of associations across studies.

Since home delivery, cold-season admission, and delayed breastfeeding were major factors considered to impact the outcomes, initiatives aimed at addressing the problem would help in reducing the prevalence of hypothermia. Community based thermal education campaigns, promotion of kangaroo mother care, and low-cost warming solutions like thermal wraps, wearable warmers, and portable incubators may all help reduce hypothermia risk. Seasonal warming methods in delivery rooms and neonatal intensive care units could help to reduce cold-season risk.

Policymakers must prioritize neonatal health within broader maternal and child health strategies. Investment in healthcare infrastructure, particularly in rural areas, is crucial to ensure that all births occur in adequately equipped facilities. This includes ensuring that healthcare workers are trained to recognize and manage hypothermia effectively.

This review’s limitations include high between-study heterogeneity, predominance of NICU-based populations, low power to identify publication bias, and a lack of severity-specific reporting. Despite these limits, sensitivity analysis indicates that the pooled prevalence estimates are robust.

## 5. Conclusions

Neonatal hypothermia is a serious public health issue in sub-Saharan Africa. It has major effects on the health and survival of newborns. This review highlights the urgent need for focused actions and policies to tackle the risks of hypothermia. The considerable heterogeneity observed across studies suggests that variations in methodology, population demographics, and local environmental contexts significantly influence reported prevalence rates, complicating direct comparisons and universal solutions. Although all included studies satisfied the minimum JBI quality levels, study quality differences may have contributed to between-study heterogeneity. Lower-quality studies often used retrospective data or less consistent temperature measurements, potentially increasing prevalence estimates. However sensitivity analyses that excluded lower-quality studies did not significantly affect the pooled results, indicating reasonable robustness. In light of the variability in study designs, population definitions and temperature measurement practices, future research should prioritize standardized neonatal hypothermia definitions, uniform severity classification and harmonized reporting frameworks.

To turn this need into action, more research is necessary. Key areas to prioritize include examining how effective specific interventions are in reducing hypothermia in various real-life situations and conducting studies over time to grasp the long-term effects of hypothermia on child development. Additionally, exploring how community health workers can provide education and support for newborn thermal care could lead to effective community-based solutions.

Ultimately, reducing neonatal hypothermia requires a two-part approach. First, we need to improve evidence-based thermal care practices at every stage of care. Second, we must raise community awareness. Achieving these goals depends on ongoing teamwork among healthcare providers, policymakers, researchers, and communities. Such cooperation is essential for building a supportive environment for every newborn and ensuring their health and survival during those critical first days of life.

## Figures and Tables

**Table 1:** Characteristics of included studies.

No	Authors	Year	Country	Study Type	Definition	Population	Sample Size	Prevalence	Risk Factors	Ref.
1.	Getaneh FB et al.	2022	Ethiopia	Cross-sectional	Auxiliary temperature < 36.5	Preterm neonates	398	83.2	1, 2, 3, 4, 5	[23]
2.	Fenta B et al.	2023	Ethiopia	Cross-sectional	Auxiliary temperature < 36.5	Preterm neonates	423	62.9	5, 6, 7, 8, 9	[24]
3.	Feyisa GT et al.	2024	Ethiopia	Cross-sectional	Auxiliary temperature < 36.5	Neonates	682	51.8	10, 11, 12	[25]
4.	Ukke GG et al.	2019	Ethiopia	Cross-sectional	Auxiliary temperature < 36.5	Neonates	354	50.3	2, 6, 7, 13, 14	[26]
5.	Nebiyu S et al.	2021	Ethiopia	Cross-sectional	Auxiliary temperature < 36.5	Neonates	356	61.5	2, 5, 7, 13, 15	[27]
6.	Yitayew YA et al.	2020	Ethiopia	Cross-sectional	Auxiliary temperature < 36.5	Neonates	202	66.8	1, 5, 10, 15	[28]
7.	Bayih WA et al.	2019	Ethiopia	Cross-sectional	Auxiliary temperature < 36.5	Neonates within 6 h of delivery	403	66.3	1, 5, 6, 16, 17, 18	[29]
8.	Demissie BW et al.	2018	Ethiopia	Cross-sectional	Auxiliary temperature < 36.5	Neonates	356	64	1, 5, 7, 15	[17]
9.	Sanni UA et al.	2024	Nigeria	Cohort	Auxiliary < 36.5	Preterm neonates	933	72.9	1, 2, 3, 15, 17	[30]
10.	Diala UM et al.	2022	Nigeria	Cross-sectional	Auxiliary < 36.5	Neonates	567	42.4	1, 2, 14, 19, 20	[31]
11.	Akida M et al.	2022	Tanzania	Cross-sectional	Auxiliary temperature < 36.5	Neonates	270	25.6	5, 13, 21	[32]
12.	Fedine U et al.	2021	Rwanda	Cross-sectional	Auxiliary temperature < 36.5	Neonates	1021	27	1, 17	[33]
13.	Phoya F et al.	2020	Malawi	Cross-sectional	Auxiliary temperature < 36.5	Neonates	112	77	2, 5, 7	[34]
14.	Nyandiko WM et al.	2021	Kenya	Cohort	Auxiliary temperature < 36.5	Neonates in the first 24 h	372	73.7	1, 7, 22, 23	[35]
15.	Mukunya D et al.	2021	Uganda	Cross-sectional	Auxiliary temperature < 36.5	Neonates	1330	51	2, 3, 7	[36]
16.	Pellegrino J et al.	2023	Ghana	Cross-sectional	Auxiliary temperature < 36.5	Low-birth-weight neonates	254	44.5	2, 5, 24	[10]
17.	Abdul-Mumin A et al.	2024	Ghana	Cross-sectional	Auxiliary < 36.5	Neonates	2469	54.76	1, 2, 25, 26	[37]
18.	Ntuli TS et al.	2023	South Africa	Retrospective descriptive study	Auxiliary temperature < 36.5	Preterm low-birth-weight neonates	252	35	14, 15, 27, 28	[38]
19.	Tshehla RM et al.	2023	South Africa	Cross-sectional	Auxiliary temperature < 36.5	Neonates	579	66	2, 8, 9, 29, 30	[39]
20.	Patel M et al.	2022	South Africa	Retrospective descriptive study	Auxiliary temperature < 36.5	Neonates born through caesarean section	1017	41	9, 28, 31, 32	[40]
21.	Ng’eny JC et al.	2020	South Africa	Cross-sectional	Skin temperature < 36.5	Low-birth-weight neonates	453	46.1	2, 15	[41]

Note: 1. Preterm birth, 2. Low birth weight, 3. Home delivery, 4. Being thrombocytopenic, 5. No skin-to-skin contact with mother/Lack of kangaroo mother care, 6. Obstetric complications during delivery, 7. Delayed initiation of breast feeding, 8. Low APGAR score, 9. Caesarian section delivery, 10. Nighttime delivery, 11. Neonates who were given traditional medication (Amessa), 12. Placing a cold object near babies’ head, 13. Early bathing, 14. Admissions during cold season, 15. Lack of resuscitation, 16. Not wearing a cap, 17. No warm intra-facility transportation, 18. Neonatal health problem, 19. Maternal age, 20. Maternal education level, 21. Early neonatal weighing, 22. Inappropriate thermal appliance, 23. Inadequate clothing, 24. Mixed feeding, 25. Meconium aspiration syndrome, 26. Birth asphyxia, 27. Use of synchronized inspiratory positive airway pressure (SiPAP), 28. respiratory distress syndrome, 29. Admission from theatre, 30. Admission to NICU, 31. Having had CPR, 32. An elevated lactate.

**Table 2:** Summary of subgroup analysis of prevalence of neonatal hypothermia.

Subgroups	No. of Studies	ES (95% CI)	Weight (%)	Heterogeneity	
				I^2^	*p*-Value
**Country**					
Ethiopia	8	63.38 (56.23, 70.53)	38.10	94.7	<0.0001
South Africa	4	47.10 (33.93, 60.27)	19.05	97.5	<0.0001
Ghana	2	50.05 (40.03, 60.08)	9.52	89.8	<0.0001
Nigeria	2	57.69 (27.80, 87.58)	9.52	99.3	0.0002
Kenya	1	73.70 (69.23, 78.17)	4.76	-	-
Malawi	1	77.00 (69.71, 84.79)	4.76	-	-
Rwanda	1	27.00 (24.28, 29.72)	4.76	-	-
Tanzania	1	25.60 (20.39, 30.81)	4.76	-	-
Uganda	1	51.00 (48.31, 53.69)	4.76	-	-
**Study design**					
Cross-sectional	17	55.30 (47.89, 62.71)	81	98.4	<0.0001
Cohort	2	73.13 (70.73, 75.54)	9.5	0	<0.0001
Descriptive	2	38.58 (32.78, 44.33)	9.5	68.3	<0.0001
**Study population**					
Neonates	14	55.48 (47.29, 63.67)	66.7	98.4	<0.0001
Preterm neonates	3	73.07 (61.67, 84.46)	14.28	96.6	<0.0001
Low-birth-weight neonates	2	45.52 (41.85, 49.19)	9.5	0	<0.0001
Neonates born through caesarean section	1	41.00 (37.98, 44.02)	4.76	-	-
Preterm low-birth-weight neonates	1	35.00 (29.11, 40.89)	4.76	-	-

**Table 3:** Meta-analysis of factors associated with neonatal hypothermia.

No.	Subgroups	No. of Studies	OR (95% CI)	Heterogeneity	
				I^2^	*p*-Value
1	Preterm birth	9	3.49 (1.98, 6.16)	97.54	<0.0001
2	Low birth weight	11	3.56 (2.36, 5.39)	79.78	<0.0001
3	No skin-to-skin contact	9	1.31 (0.55, 3.13)	94.19	=0.5357
4	Lack of resuscitation	6	2.56 (1.75, 3.76)	49.7	<0.0001
5	Delayed initiation of breastfeeding	6	2.38 (1.57, 3.61)	81	<0.0001
6	Admission during the cold season	3	1.80(1.33, 2.44)	0	=0.0002
7	Home delivery	3	1.94 (1.51, 2.50)	0	<0.0001
8	Early bathing	3	3.03 (0.98, 9.38)	85.59	=0.0546

**Table 4:** Summary of risk factors associated with hypothermia in sub-Saharan Africa.

Domain	Risk Factors	Direction of Association	Pooled OR (95% CI)	Strength of Evidence
Physiological	Preterm birth	Increased	3.49 (1.98, 6.16)	Strong
	Low birth weight	Increased	3.56 (2.36, 5.39)	Strong
Behavioral	No skin-to-skin contact	Increased	1.31 (0.55, 3.13)	Weak
	Lack of resuscitation	Increased	2.56 (1.75, 3.76)	Moderate
	Delayed initiation of breastfeeding	Increased	2.38 (1.57, 3.61)	Moderate
	Early bathing	Increased	3.03 (0.98, 9.38)	Limited
Environmental	Admission during the cold season	Increased	1.80(1.33, 2.44)	Moderate
Delivery related	Home delivery	Increased	1.94 (1.51, 2.50)	Moderate

## Data Availability

Data are contained within the article. No new data were created or analyzed in this study.

## Supplementary Materials

The following supporting information can be downloaded at: https://www.mdpi.com/article/10.3390/jcm15051818/s1, Table S1: PRISMA checklist; Figure S1: Forest plot preterm birth; Figure S2: Forest plot low birth weight; Figure S3: Forest plot no skin-to-skin contact with mother; Figure S4: Forest plot lack of resuscitation; Figure S5: Forest plot delayed initiation of breast feeding; Figure S6: Forest plot admissions during cold season; Figure S7: Forest plot home delivery; Figure S8: Forest plot early bathing.

## Abbreviations

The following abbreviations are used in this manuscript:

PRISMA	Preferred Reporting Items for Systematic Review and Meta-Analysis statement
JBI	Joanna Briggs Institute
OR	Odds ratio
CI	Confidence interval
WHO	World Health Organization
APGAR	Appearance, Pulse, Grimace, Activity, and Respiration
SiCPAP	Synchronized inspiratory positive airway pressure
NICU	Neonatal intensive care unit
CPR	Cardiopulmonary resuscitation
ES	Effect size
