# Supplementary figures and images for: Prevalence and Factors Associated with Neonatal Hypothermia in Sub-Saharan Africa: Systematic Review and Meta-Analysis
Source: J Clin Med. 2026 Feb 27;15(5):1818. doi: 10.3390/jcm15051818 (PMC12985583; doi:10.3390/jcm15051818)

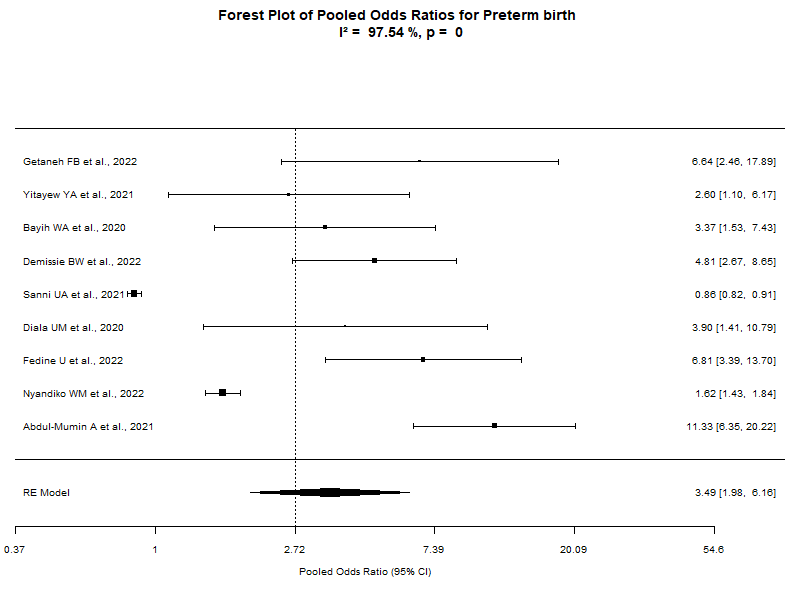

Supplement: Supplementary file 1 [file jcm-15-01818-s001.zip › Figure S1 forest_plot_Preterm_birth.png]

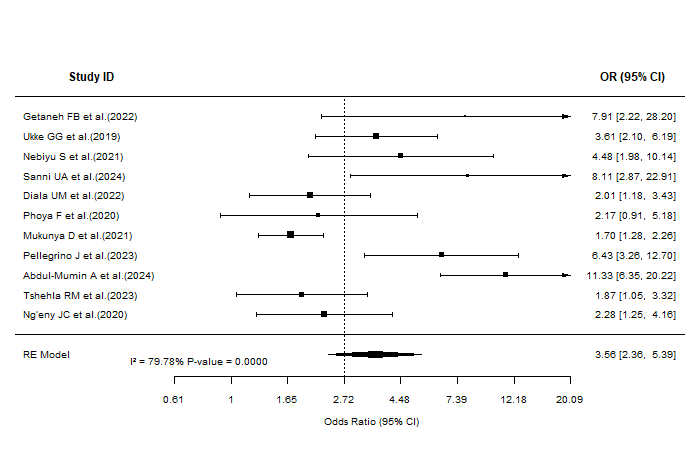

Supplement: Supplementary file 1 [file jcm-15-01818-s001.zip › Figure S2 forest_plot_Low_birth_weight.png]

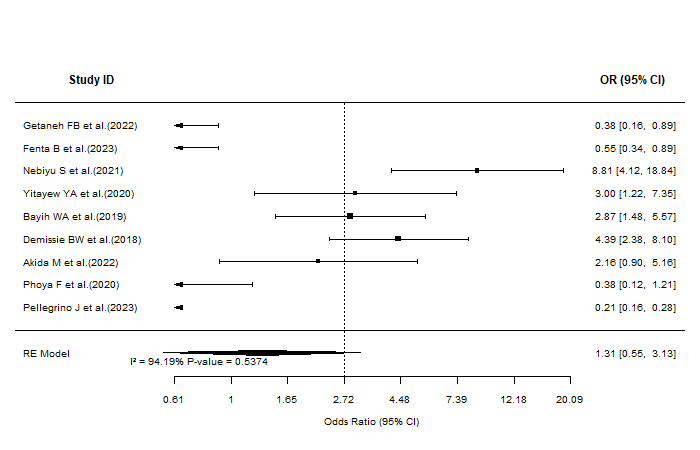

Supplement: Supplementary file 1 [file jcm-15-01818-s001.zip › Figure S3 forest_plot_No_skin-to-skin_contact_with_mother.png]

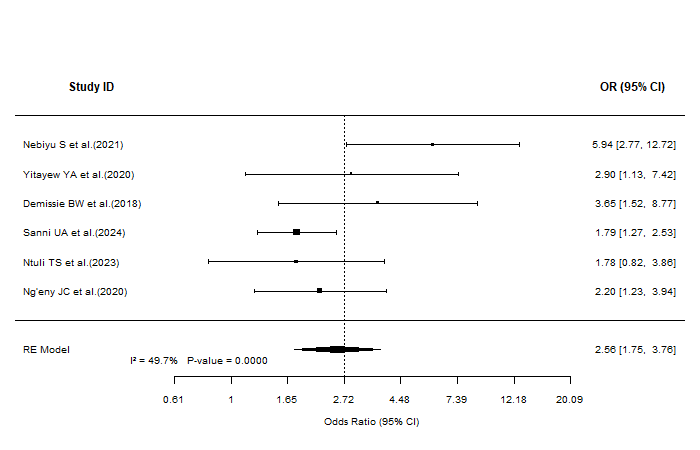

Supplement: Supplementary file 1 [file jcm-15-01818-s001.zip › Figure S4 forest_plot_Lack_of_resuscitation.png]

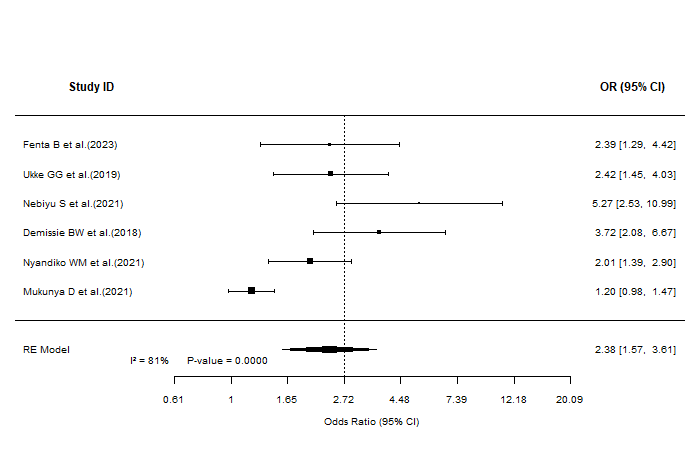

Supplement: Supplementary file 1 [file jcm-15-01818-s001.zip › Figure S5 forest_plot_Delayed_initiation_of_breast_feeding.png]

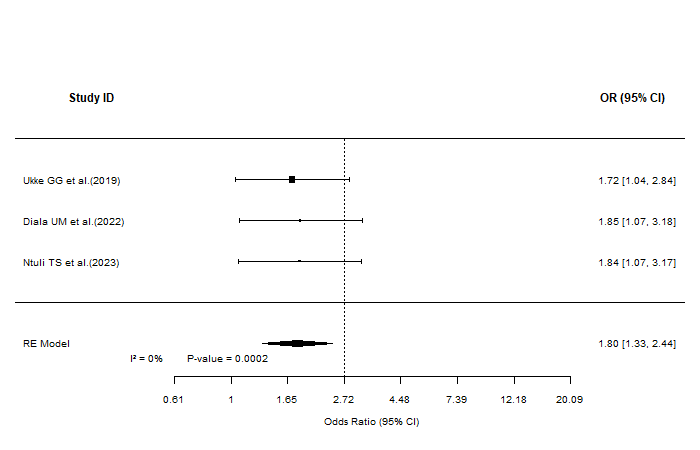

Supplement: Supplementary file 1 [file jcm-15-01818-s001.zip › Figure S6 forest_plot_Admissions_during_cold_season.png]

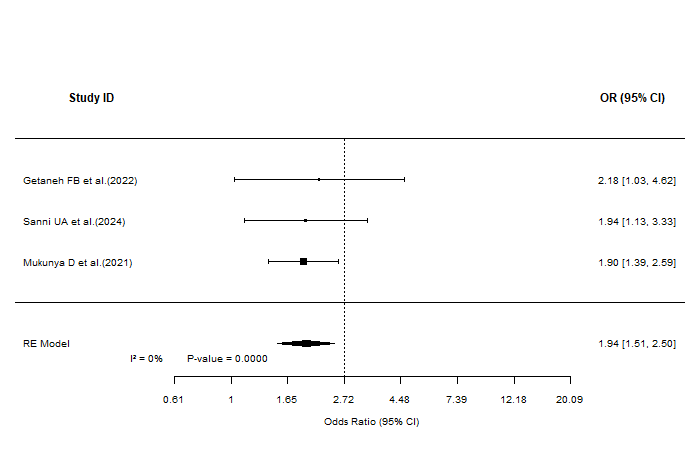

Supplement: Supplementary file 1 [file jcm-15-01818-s001.zip › Figure S7 forest_plot_Home_delivery.png]

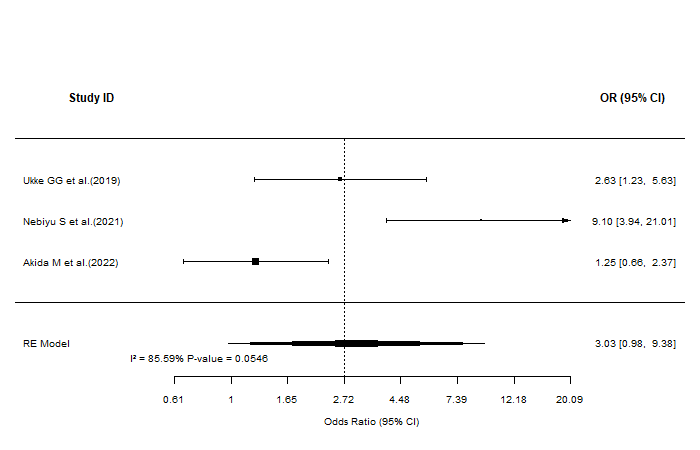

Supplement: Supplementary file 1 [file jcm-15-01818-s001.zip › Figure S8 forest_plot_Early_Bathing.png]
